# Experimental Artifacts for Morphological Tweaking of Chemical Sensor Materials: Studies on ZnO

**DOI:** 10.3390/s120608259

**Published:** 2012-06-13

**Authors:** Ikram Ul Haq, Abdul-Majeed Azad

**Affiliations:** 1 National Center of Excellence in Physical Chemistry, University of Peshawar, Peshawar 25120, Khyber Pakhtunkhwa, Pakistan; 2 Department of Chemical Engineering, The University of Toledo, 2801 W. Bancroft St., Toledo, OH 43606, USA; E-Mail: abdul-majeed.azad@utoledo.edu

**Keywords:** homogeneous precipitation, urea, hexamethyltetraamine, monosized particles, monodispersion, zinc oxide, gas sensor

## Abstract

Sensing mechanisms of gases on solid structures are predominantly surface-dominated. Benign surface features in terms of small grain size, high aspect ratio, large surface area and open and connected porosity, are required to realize a successful sensor material. Such morphological artifacts are a function of the fabrication and processing techniques employed. In this paper, we describe the fabrication of monoshaped and monosized zinc oxide (ZnO) particles by a homogeneous precipitation method, using urea and/or hexmethyltetraamine as the reductant. The effect of operating conditions and experimental variables, such as the relative concentration of the precursors, temperature, and the aging time on the morphology of the resulting particles was studied systematically. These experimental parameters were optimized in order to achieve particles of uniform morphology and of narrow size distribution. Some of these particles were employed for the detection of ammonia gas at room temperature.

## 1. Introduction

One aspect of current interest and great relevance to the fundamental understanding of the behavior of materials is the role of dimensionality and size on their optical, chemical and mechanical properties for application in a wide range of devices. It has now been well-established that in the lower range of particle size, one-dimensional systems exhibit novel physical and chemical properties that can be exploited, for example, in optics, catalysis and photocatalysis, lab-on-a-chip sensors/detectors and electrocatalysts, to name but a few. Hence, such systems are being synthesized and studied in great detail and thus they become model systems to study and correlate the theoretical explanations that are still in progress. Such behavior is almost nonexistent in the bulk material where the particle size is relatively large; this propels the desire to introduce these attributes in functional materials.

One area where such features in the materials are of immense relevance is the field of solid-state chemical sensors. Work on sensor development is being carried out worldwide in industry, academia, and government facilities. This is driven by the need for improved, fast, *in-situ* and online detection and quantification of various gaseous pollutants in a host of industries including power generation, automobile, chemical, pharmaceutical, electronics, biomedical, health and environmental protection and preservation. The global market for advanced materials and sensors increased from $5.7 billion in 2006 to $6.8 billion in 2007 and is estimated to reach $9.4 billion by 2012, a compound annual growth rate (CAGR) of 6.9%. The global market for automotive sensor technologies increased from $7.3 billion in 2006 to $8.0 billion in 2007 and is estimated to reach $13.5 billion by 2012, a CAGR of 10.8%. The market for chemical process monitoring devices was $50.9 billion in 2006 and at a CAGR of 4%, the market will surpass $62 billion by the end of 2012. Demand for chemical sensors in the US alone is forecast to reach $5 billion in 2012, buoyed by continued strong demand for biosensor products in the medical and diagnostic industries, and increased spending on public safety and security in the face of a potential biological or chemical terrorist attack. Other markets such as environmental monitoring and industrial processing are also projected to experience healthy gains, though not as fast as medical and diagnostics; for example, the global environmental sensor and monitoring market was worth $11.3 billion in 2011. This market is expected to reach $15.3 billion in 2016 with a 6.5% CAGR in 5 years [1].

Detection of chemical species and their quantification have become important tasks in many industrial applications. High selectivity, enhanced sensitivity and short response time are some of the key features sought in these devices. Since the sensing mechanism and catalytic activity of these chemical sensors are largely microstructure-dominated, features such as small grain size, high aspect ratio, large surface area, and, open/connected porosity are required to realize a successful sensor material [2–4]. Some routinely-employed methods to achieve small grains and exotic morphologies in potential semiconducting sensor materials are sol-gel and other low temperature solution routes.

In this context, semiconducting metal-oxide based gas sensors are very popular and versatile [5]. The gas sensing properties of these sensors stem from the fact that reversible adsorption/desorption phenomenon of gases species on the metal oxide surfaces produces measurable changes in their electrical properties, such as the resistance [6]. The magnitude of resistance change is generally proportional to some function of the concentration of the gases species in question. In recent years, ZnO has been investigated extensively due partly to the fact that in addition to being a simple oxide that is quite inexpensive and available in relative abundance, it is also an ideal semiconductor with a band gap of 3.37 eV [7]. In fact, the history of ZnO-based gas sensors can be traced back to as many as 60 years [8]. Moreover, ZnO particles can be synthesized in a variety of ways in different shapes and sizes [9–12]. ZnO-based gas sensors have been produced and used in different geometries for the detection of various types of gases of environmental concern [13–19].

Ammonia is commercially produced and widely used in many common industrial processes, such as food processing, fertilizers and organic amines, etc. It is also a toxic gaseous pollutant [20–25]. In many of these processes, there always exist dangers of ammonia leakage. Hence, early detection of ammonia prior to an accident is warranted. In this regard, development of efficient, durable, and stable ammonia gas sensors that operate at or near room temperatures, is highly desirable [26]. A number of parameters, such as operating temperature, humidity, interfering gases, etc., affect the performance and sensitivity of the sensor [27].

Generally, the semiconducting oxide-based sensors operate at temperatures higher than ∼200 °C; this limits their use in the lower temperature regime. However, a number of attempts have been made to produce ZnO-based ammonia sensors that operate at room temperature [28–31].

In addition, particle shape, size, and their uniformity play a vital role in determining and controlling the sensing behavior of ZnO. Thus, there exists ample room for further improvisation in this important area, especially in order to tailor the performance and sensitivity of ZnO based sensors, by manufacturing ZnO particles of different morphological features.

Historically, ZnO particles have been prepared by various routes, such as the electrophoretic deposition [11], hydrothermal synthesis [32], thermal evaporation [33], chemical vapor deposition [34], homogeneous precipitation [35], refluxing route [36], and thermal annealing processes [37]. Among these, the urea-based homogeneous precipitation method is considered the most versatile and has been used extensively by many for the synthesis of monosized and monoshaped particles [38–43]. Use of hexamethyltetraamine (HMTA) as an effective reductant to produce hexagonal ZnO rods of various lengths and cross-sections from aqueous solutions of zinc precursors is also quite effective [44]. Slow evaporation of solid ZnO powder also generates exotic nanostructures in high purity [45].

In the light of the ease of preparation and assembly as a sensor device, the present work outlines the facile synthesis of uniform particles of zinc oxide by the urea- and HTMA-based homogeneous precipitation routes. In these preparatory methods, the reactant solutions are heated either to boiling under reflux conditions or to the near-boiling range. In both cases, precipitated particles of either zinc oxide or zinc basic carbonate of unique morphologies are formed. One of these batches of zinc oxide particles was employed to fabricate sensor elements for the detection of ammonia vapors at room temperature.

## 2. Experimental

### 2.1. Materials

Analytical reagent grade zinc nitrate (Sigma), urea (Sigma), hexmethylenetetraamine (Alfa-Aesar) and ethylene glycol (Sigma) were employed as the starting chemicals and used without further purification. The stock and working solutions were made in deionized water; any suspended impurities were removed by filtration through a membrane filter.

### 2.2. Particles Preparation

Particles of zinc compounds were prepared by heating different batches of aqueous solutions, composed of urea or hexamine (0.04–0.6 mol/L), zinc nitrate hexahydrate (0.01–0.05 mol/L), and ethylene glycol (4–15 vol.%) at different temperatures (80–95 °C) for various periods of time (30–150 min) in a thermostatic water bath or heated to boiling under reflux in a 1L round bottom flask using a heating mantle. In case of precipitation using a water bath, the bath temperature was adjusted to the desired value before lowering the reactor (a 300 mL Pyrex glass vessel equipped with a Teflon coated stopper) in it. Similarly, in reflux experiments, either the reactant solutions were prepared at room temperature and then subjected to boiling or urea solution was boiled separately and then added to the solution containing zinc nitrate and/or zinc nitrate + ethylene glycol. In both methods, the precipitated particles were isolated from solution by vacuum filtration through a membrane filter, washed first with water and then with ethanol. The obtained solids were transferred to desiccator for drying.

### 2.3. Characterization

The crystallinity of the precipitated particles was confirmed from the X-ray diffraction patterns collected on a JEOL JDX-3532 machine using monochromatic CuK_α_ radiations in the range of 10–80° (2-θ) in the steps of 0.05°, operated at a voltage of 40 kV and 20 mA current. The microstructural aspects of the precipitated solids were examined with a scanning electron microscope (JEOL, JSM-5910) operating at the accelerating voltage of 15 keV. Prior to microscopic imaging, the powder samples were mounted on an aluminum stub using a carbon conducting tape and sputtered with gold in a vacuum chamber (JEOL, JFC-1600, Auto Fine Coater). The thermogravimetric analysis (TGA) and differential thermal analysis (DTA) of the samples were carried out by using the Diamond TG/DTA analyzer from Perkin Elmer. The samples were heated from room temperature to 800 °C at a rate of 5° min^−1^ in static air. The infrared spectroscopic analyses were performed with an FTIR (Shimadzu, IR Prestige-21, FTIR-8400S) instrument in the 4,000–400 cm^−1^ wave number range. A programmable furnace was used for the calcination of the as-precipitated samples using a heating rate of 5° min^−1^. The samples were cooled to room temperature by natural cooling after switching off the furnace power.

### 2.4. Sensor Fabrication and Response Measurements

A slurry of ZnO powder in ethylene glycol was prepared and applied smoothly on the sensor platform (5 mm × 5 mm alumina plate decorated with interdigitated gold electrodes) in the form of a uniform layer (∼100 μm thick). The sensor film was dried at 60 °C for 2 h. Gold lead wires (0.25 mm diameter, Alfa-Aesar) were attached to the contact pads via silver paste which was cured in three different stages between room temperature and 350 °C so as to form good ohmic contacts. The ends of the gold wires were connected to a high impedance Agilent 34220A digital multimeter, which in turn was connected to a desktop PC via an interface card. Sensor resistance data was acquired and displayed in real-time with the help of a commercial software.

Gas sensing experiments were carried out by exposing the sensor to the test gas, either in the flowing or the static mode. In the static mode, ammonium hydroxide solutions (0.25%, 2.5%, 6%, 15% and 25%) were placed in 25 mL capacity Pyrex glass vials and the vials were tightly closed, leaving equal headspace above each solution. Concentration of ammonia vapors above the ammonium hydroxide solutions in the headspace in the vials was 590, 5190, 11,000, 35,000 and 120,000 ppm, respectively. The sensor was exposed to the headspace in each vial for a given period of time at the ambient temperature, and the sensor resistance was continuously measured. In the dynamic mode, two identical sensor assemblies were connected in series in such a way that the outlet port of the first functioned as the inlet of the second sensor. Same stream of the test gas was passed through both the set-ups and the variation in sensor resistance was continuously monitored in real-time.

## 3. Results and Discussion

### 3.1. Precipitation

**Figure 1.**
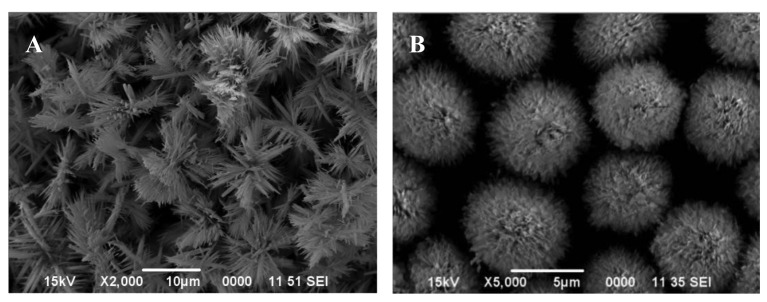
SEM images of the particles precipitated from an aqueous solution containing 0.4 mol/L urea and 0.035 mol/L zinc nitrate with 10 vol.% ethylene glycol, heated for 45 min. at: (**A**) 80 °C and (**B**) 95 °C.

Based on our previous work on the fabrication of metal hydroxide carbonate and/or metal oxide particles by the urea-based homogeneous precipitation process [38,46,47], attempts were made to produce uniform particles of zinc oxide as well. For this, aqueous solutions comprising urea and zinc nitrate with and without ethylene glycol or ethanol of known concentration were heated in the temperature range of 80–95 °C for duration ranging between 30 and 150 min. without stirring. Heating initiated the decomposition of dissolved urea, leading to the release of carbonate and hydroxyl ions in the aqueous medium. Once their concentration reached an optimum level, they react with the zinc ions in solution, resulting in the formation of a turbid dispersion (white precipitate). Systematic SEM imagery of these particles reveal that the experimental conditions, such as the relative composition of the starting solutions, temperature, and the aging time, affect the morphology of the precipitated particles significantly. Figure 1 shows the morphology of the particles from solutions of same compositions precipitated at 80 and 95 °C, illustrating the marked effect of temperature.

It appears that the formation of fully grown particles by the aggregation process from the initially formed nuclei is highly temperature dependent, resulting in dendrites (A) at lower and spheroids (B) at higher temperature.

**Figure 2.**
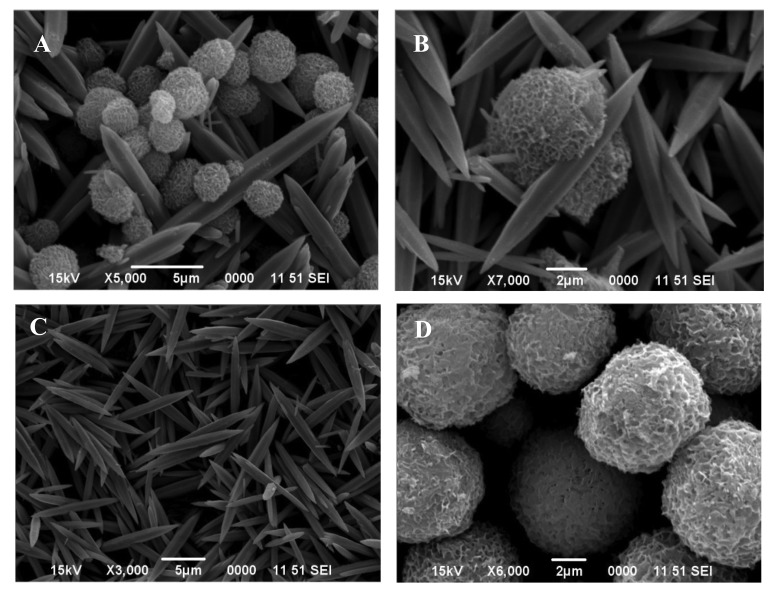
SEM images of the particles precipitated from a mixture, containing 0.4 mol/L urea, 0.035 mol/L zinc nitrate and 10 vol.% ethylene glycol. The mixture was heated to boiling under reflux for: 50 min (**A** and **B**) and 20 min (**C**). (**D)** obtained by reflux boiling of solution C for 30 min.

**Figure 3.**
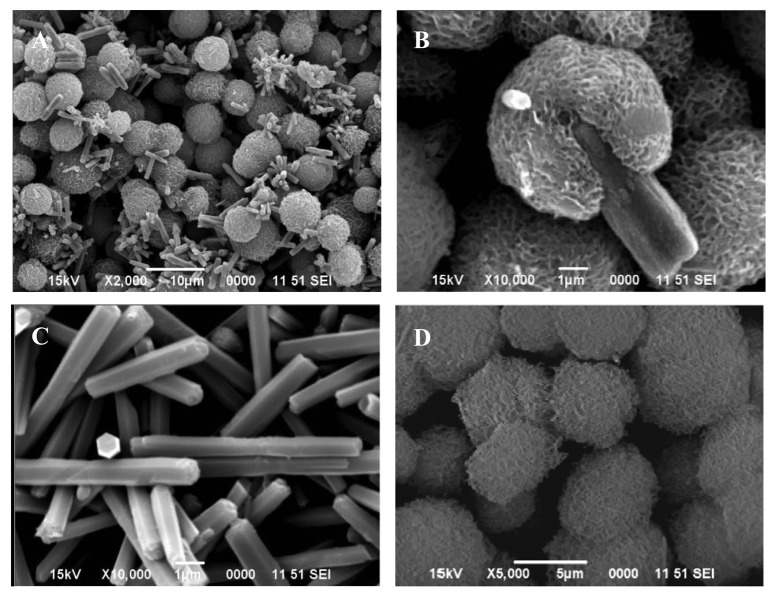
SEM images of the particles precipitated from a mixture containing 0.4 mol/L urea and 0.035 mol/L zinc nitrate. The mixture was heated to boiling under reflux for: 50 min (**A** and **B**), and 20 min (**C**). (**D**) obtained by reflux boiling of solution C for 30 min.

The crucial role of temperature in the precipitation process and its effect on the morphological artifacts was further evaluated by a different experimental strategy. In this case, the 0.4 mol/L urea solution and 0.035 mol/L zinc nitrate solution were mixed together with and without 10 vol.% ethylene glycol, and heated until boiling for 1h under reflux. The resulting precipitates in the two instances were separated and dried. The SEM images of the particles formed in the two cases are shown in Figure 2 (with ethylene glycol) and Figure 3 (without ethylene glycol). The particles formed in the presence of ethylene glycol were composed of: (i) either a mixture of spheroidal and sharp-tipped symmetrical needles; (ii) only needles or (iii) only spheroids, depending upon the duration over which reflux was carried out. The particles formed in the absence of ethylene glycol were mostly spherical and rod-structured (Figure 3). A comparison of the morphological features illustrated in Figures 2 and 3, clearly indicates that ethylene glycol has likely functioned as some sort of ‘shape modifying’ additive, especially for the non-spherical particles. Furthermore, this difference is indicative of different nucleation steps operative in the two cases, resulting in the formation of particles in bimodal distribution.

**Figure 4.**
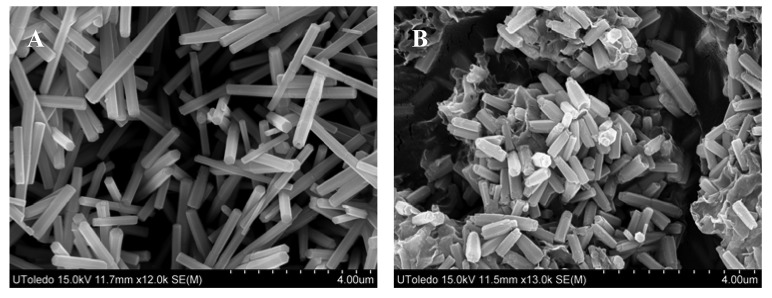
SEM images of the particles obtained by reflux boiling of aqueous solution containing 0.035 mol/L zinc nitrate and 0.17 mol/L urea for: (**A**) 20 min. and (**B**) 5 min.

The effect of urea concentration on the particle morphology was also studied. It was found that varying urea concentration had marked effect on the particle size, without changing their morphological features. Figure 4(A) is the SEM image obtained on particles made from a mixture containing 0.17 mol/L (instead of 0.4 mol/L) of urea, without changing the zinc nitrate concentration which was kept constant at 0.035 mol/L. Comparison of Figure 4(A) with 3(C) shows that the use of lower concentration of urea with same reflux time (20 min) led to the formation of relatively smaller particles. Furthermore, the particles formed after 5 min. of refluxing (Figure 4(B)) compared to 20 min. (Figure 4(A)) were much smaller and in aggregates, clearly illustrating that these particles were in their initial stages of growth.

**Figure 5.**
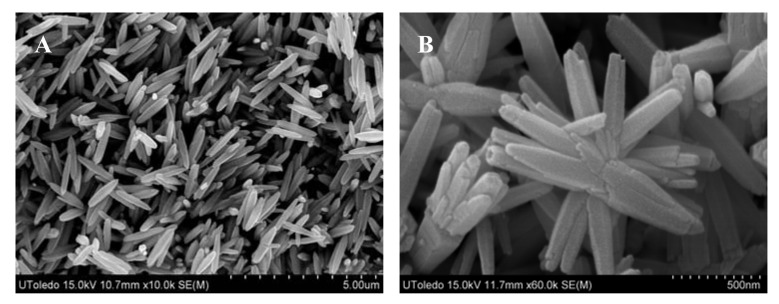
SEM images of the particles produced by reflux boiling of aqueous solution containing 0.034 mol/L zinc nitrate and 0.013 mol/L hexamine for 40 min. with: (**A**) 4.5 vol.% ethylene glycol and (**B**) ethanol.

In order to discern the effect of different reducing/precipitating agent on the particle characteristics, hexmethylenetetraamine was used instead of urea, in the presence of ethylene glycol in one case and ethanol in the other. The SEM images on the particles precipitated from solutions containing 0.013 mol/L hexamine and 0.034 mol/L zinc nitrate with 4.5 vol.% of ethylene glycol, are shown in Figure 5(A), while those in the presence of ethanol are shown in Figure 5(B). This led to the conclusion that the applied experimental conditions, and solution composition has significant influence on the morphology and uniformity of the resulting particles. For example, the particles obtained in the presence of ethylene glycol were more regular and uniform than those with ethanol.

**Figure 6.**
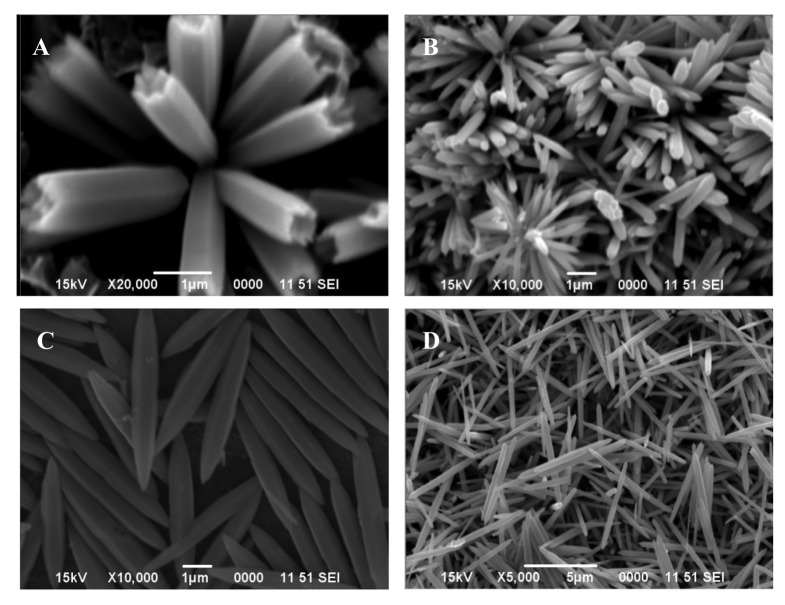

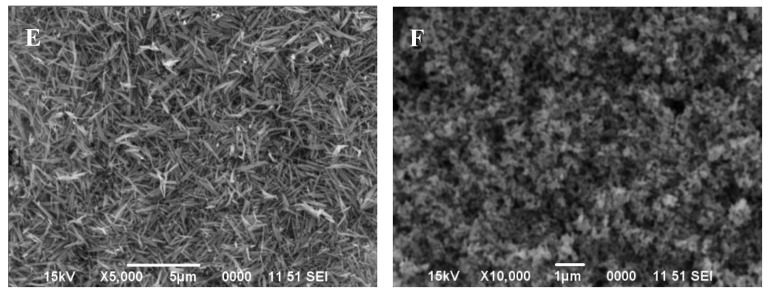
SEM images of the particles produced by reflux boiling for 30 min. the aqueous solutions containing: (**A**) 0.2 mol/L urea, and 0.03 mol/L zinc nitrate; (**B**) 0.4 mol/L urea and 0.03 mol/L zinc nitrate; (**C**) 0.4 mol/L urea and 0.03 mol/L zinc nitrate, with 15 vol.% ethylene glycol; (**D**) 0.4 mol/L urea and 0.03 mol/L zinc nitrate, with 4 vol.% ethylene glycol; (**E**) 0.4 mol/L urea and 0.015 mol/L zinc nitrate, with 4 vol.% ethylene glycol. Image (**F)** is for the particles obtained by calcining the particles in D at 700 °C for 1 h (heating rate: 15°/min.)

Using this strategy, the experimental conditions were adequately optimized in order to fabricate uniform particles in different morphologies and selected images of urea-mediated precipitates are shown in Figure 6. It is added that the optimized preparation conditions for all batches of the particles, included in this paper, have also been summarized in Table 1.

X-ray diffraction of these particles revealed that each was crystalline in nature but possessed a different chemistry. For example, the particles shown in: (i) Figure 1(A,B) are zinc basic carbonate; (ii) Figures 2(A,B), and 3(A) and 3(B)) consist of a mixture of zinc basic carbonate and zinc oxide; and (iii) Figures 2(C), 3(C), 4(A), 4(B) and 6(A–F) are pure zinc oxide.

**Figure 7.**
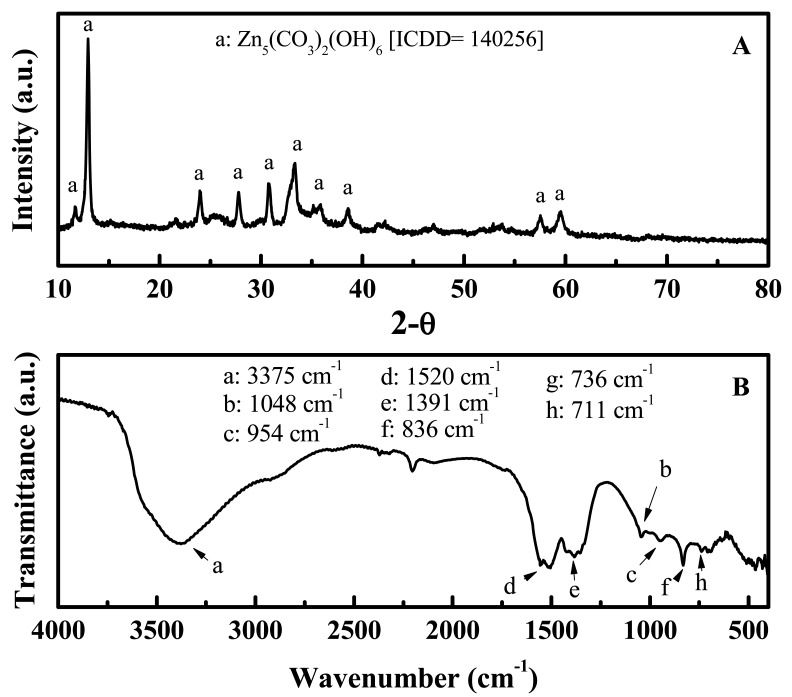
(**A**) X-ray diffraction (XRD) patterns of the Zn_5_(CO_3_)_2_OH)_6_ particles with morphology shown in Figure 1(B); (**B**) Fourier Transform Infrared (FTIR) signature of the zinc basic carbonate particles shown in Figure 1(B).

The particles whose morphology is shown in Figure 1B were used for further investigation. Their XRD pattern is shown in Figure 7(A), which corresponds to zinc basic carbonate (Zn_5_(CO_3_)_2_(OH)_6_). This was supported by their FTIR analysis carried out in range 4,000–400 cm^−1^, and shown in Figure 7(B). The absorption bands corresponding to the vibration modes characteristic of hydroxyl and carbonate groups could be easily seen. For example, the broad absorption bands around 3,375 cm^−1^ as well as at 1,048 and 954 cm^−1^ belong to the stretching vibration of hydroxyl group. The peaks at 1,520, 1,391, 836, 736 and 711 cm^−1^ are indicative of the presence of carbonate group in the precipitated solid.

**Figure 8.**
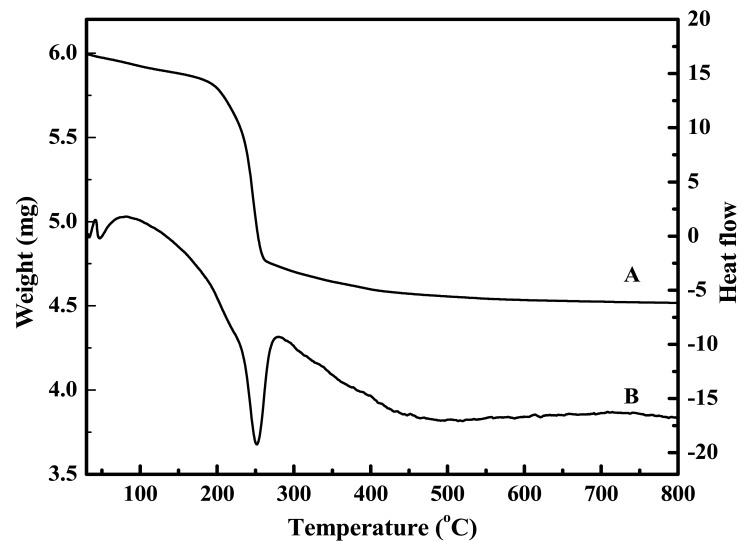
The TG (**A**) and DTA (**B**) curves of the Zn_5_(CO_3_)_2_OH)_6_ particles.

The simultaneous TG/DTA traces collected on the zinc basic carbonate particles are shown in Figure 8. Two endothermic peaks in the vicinity of 50 and 250 °C are seen on the DTA curve, while a major weight loss appears in the vicinity of 250 °C on the TG curve. These correspond to the loss of water and carbon dioxide, respectively, from the basic carbonate. Moreover, the total weight loss amounts to about ∼25% which agrees well with the theoretical weight loss (25.9%) anticipated for the following decomposition reaction:

(1) $${\text{Zn}}_{5}{{(\text{CO}}_{3})}_{2}{\left(\text{OH}\right)}_{6}\to {5\text{ZnO}+2\text{CO}}_{2}{+3\text{H}}_{2}\text{O}$$

**Figure 9.**
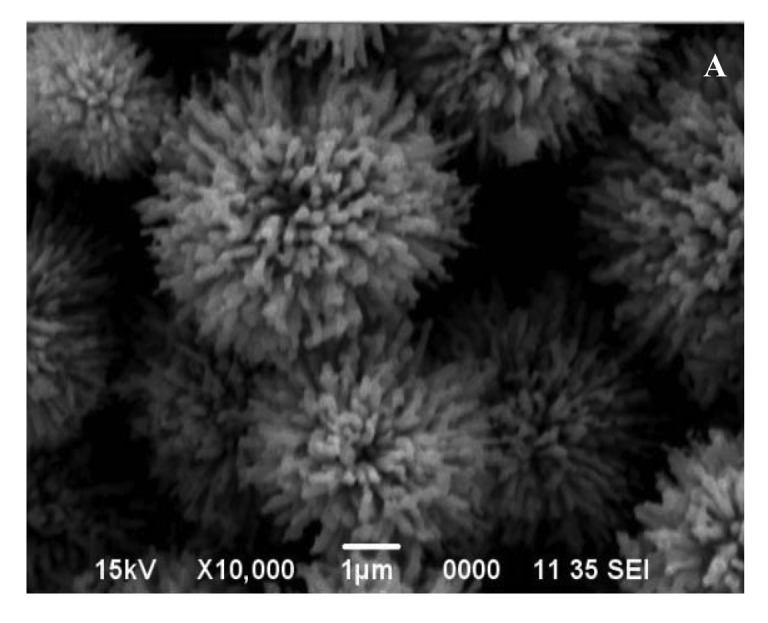

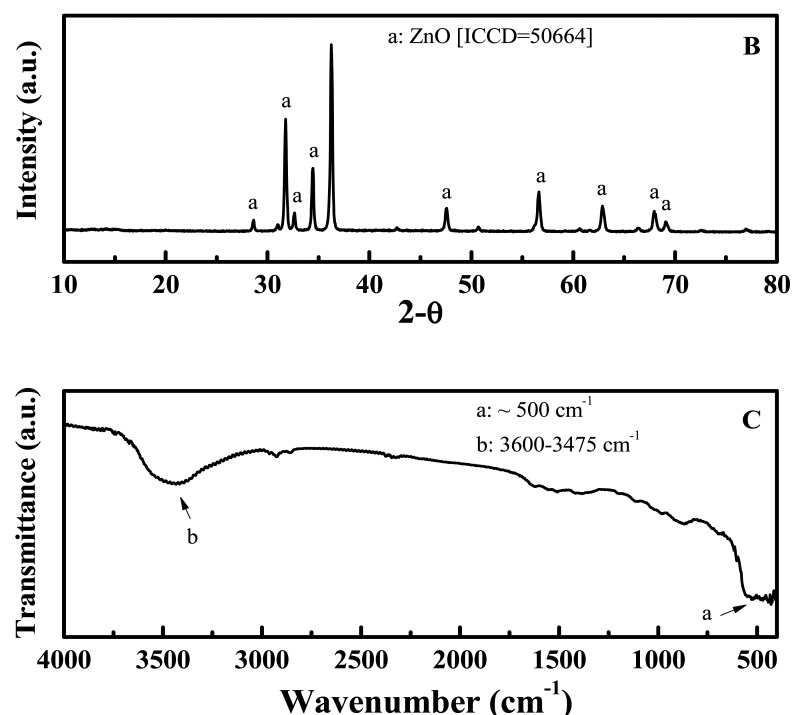
(**A**) SEM of the zinc oxide particles obtained by calcining zinc basic carbonate (1B), at 700 °C for 1 h; (**B**) XRD signature of the particles shown in 9A; (**C**) Fourier Transform Infrared (FTIR) signature collected on the zinc oxide particles shown in 9A.

The SEM image of the basic carbonate particles after calcination for 1 h at 700 °C at a ramp rate of 5° min^−1^, is shown in Figure 9(A). Comparison with Figure 1(B) shows that the overall microstructure in the calcined particles stayed almost intact, albeit with slight expected increase in the porosity. The XRD pattern shown in Figure 9(B) corresponds to that of ZnO. In the FTIR signature of the calcined particles (Figure 9(C)), with the exception of a broad absorption band in the 3,600–3,475 cm^−1^ range (corresponding to stretching vibration of hydroxyl group, probably due to adsorbed water), almost all other absorption bands have disappeared; the prominent band around ∼500 cm^−1^ is characteristic of M-O bonding, which clearly showed the formation of ZnO, as corroborated by the XRD.

### 3.2. Gas Sensing Properties

**Figure 10.**
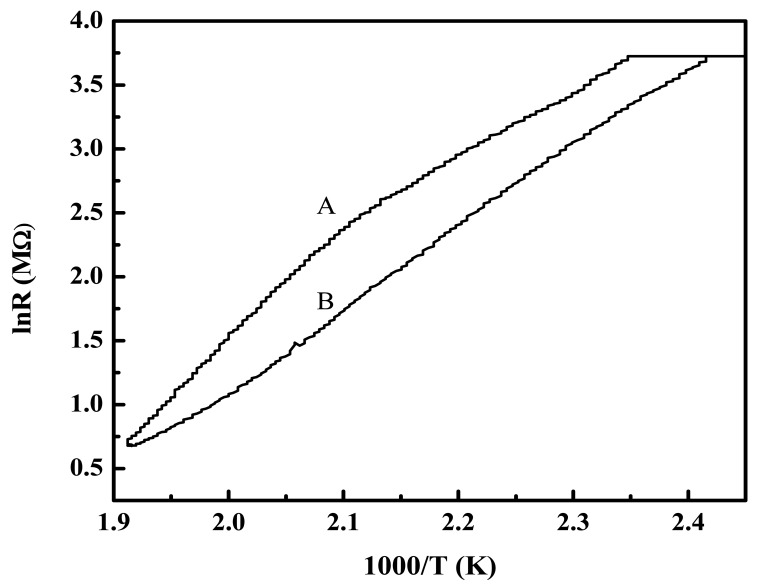
Temperature dependence of the resistance of ZnO-based sensor film. Inset caption: A: Heating mode B: Cooling mode.

Zinc oxide (ZnO) is a well-known n-type semiconductor whose resistance decreases with increase in temperature as seen from Figure 10 with slight hysteresis between the heating and cooling cycles.

#### Ammonia Sensing

**Figure 11.**
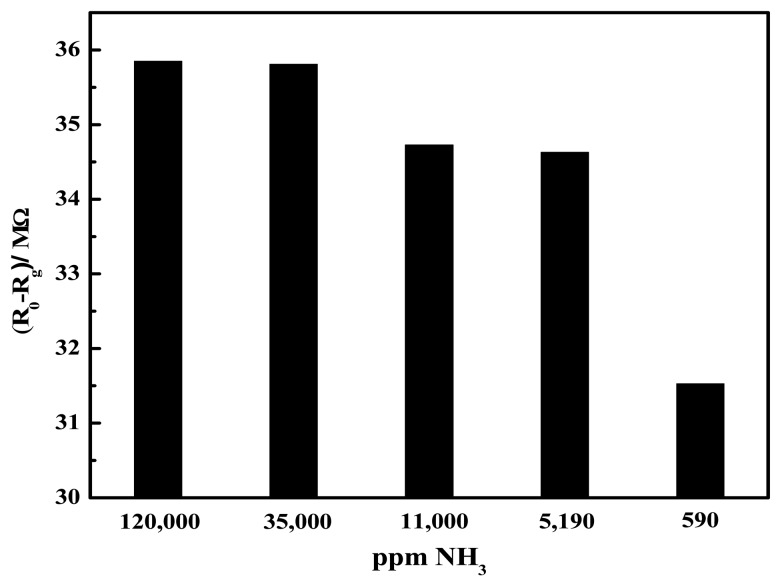
Relative change in the resistance of ZnO thick film on exposure to different levels of ammonia in air at room temperature (23 °C). R_o_ and R_g_ represent the film resistance in air and ammonia/air mixture, respectively.

**Figure 12.**
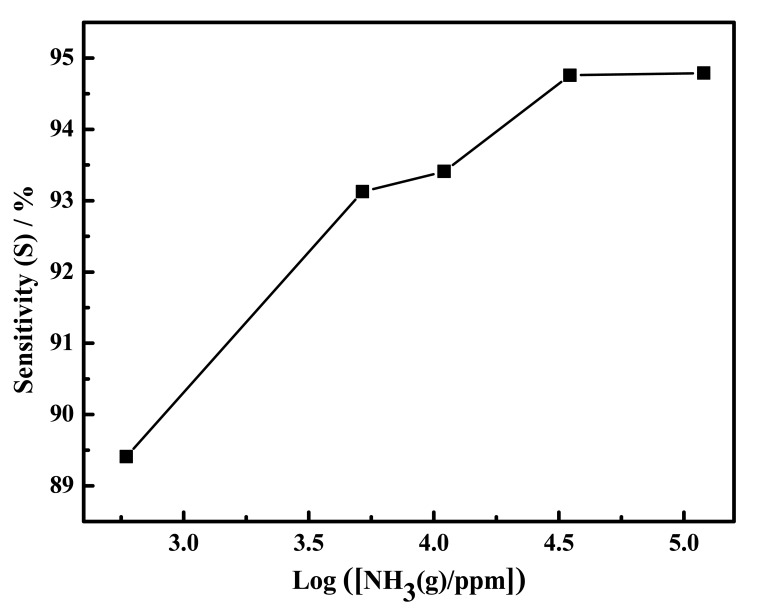
Sensitivity of the ZnO thick film as a function of ammonia concentration at room temperature (23 °C).

The response of ZnO-based sensor towards ammonia vapors present in the headspace of the ammonium hydroxide solutions, in shown in Figure 11. As can be seen, the resistance changed monotonically with ammonia concentration. The sensitivity (S) of the sensor defined as:

(2) $${\text{S}=[\Delta \text{R}/\text{R}}_{0}]\times 100$$

is shown in Figure 12, which indicates the approach of the sensing limit to saturation to be around ∼35,000 ppm of ammonia.

**Figure 13.**
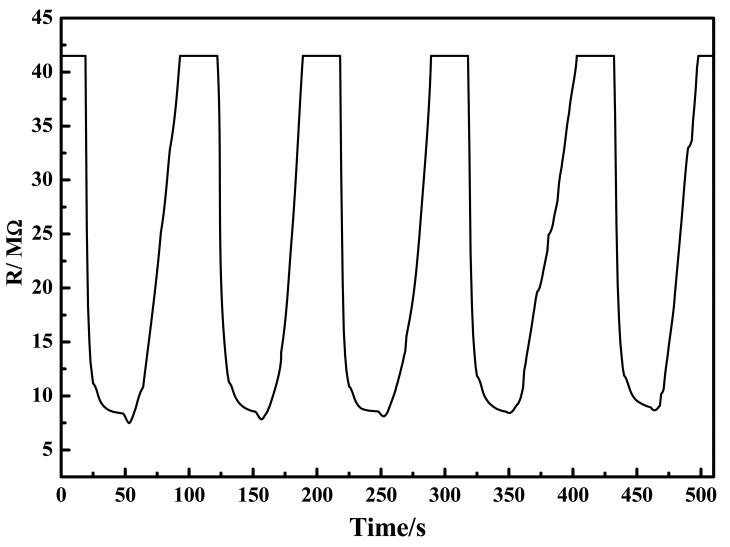
Evidence of reproducible response of the ZnO thick film to 11,000 ppm ammonia at room temperature (23 °C).

Figure 13 shows the reproducible response of the ZnO-based ammonia sensor over five cycles of alternating exposure to ammonia in the headspace and dry air. The exposure time to each of these environments was 60 s, since our preliminary measurements revealed that the sensor took this long to reached steady-state. Similar cycles were repeated weekly, each run comprising three cycles, and the dependence of resistance on the ammonia concentration was almost identical to that shown in Figure 13. These observations revealed that in addition to excellent performance reproducibility, the sensor also exhibited good shelf life. This much-desired behavior could be attributed to the particle uniformity in the ZnO powder that was produced in this work and used for sensor fabrication. Figure 13 was also used to compute, the response time (t_90_-response) and recovery time (t_90_-recovery) which turned out to be ∼14 and ∼41 s, respectively. Relatively longer recovery time could be due to a number of factors. First, it is indicative of the fact that desorption of ammonia from the ZnO surface was somewhat sluggish—a fact which is common to many of the chemical sensors in thick film format. Second, it is likely that the sensing mechanism is not based on simple adsorption-desorption phenomenon at a molecular level.

In order to account for the behavior of the sensor towards ammonia observed in Figure 13, we propose the following pathway for the interaction of ammonia with the sensor surface:

(3) $$½\;{\text{O}}_{2}\left(\text{g}\right)+{\text{e}}^{-}\;\to \;{\text{O}}_{\left(\text{ads}\right)}^{-}$$

(4) $${\text{NH}}_{3}(\text{gas})\;\to \;{\text{NH}}_{3\left(\text{ads}\right)}$$

(5) $${\text{NH}}_{3\left(\text{ads}\right)}{+\text{O}}_{\left(\text{ads}\right)}^{-}\;\to \;{{[\text{NH}}_{2}.\text{H}---\text{O}]}_{\left(\text{ads}\right)}^{-}$$

(6) $$\begin{array}{c} {2[\text{NH}}_{2}{.\text{H}---\text{O}]}_{\left(\text{ads}\right)}^{-}{+\text{O}}_{\left(\text{ads}\right)}^{-}\;\to \;{\text{N}}_{2}{(\text{g})+3\text{H}}_{2}{\text{O}+3\text{e}}^{-} \end{array}$$

First, there is a co-adsorption of gaseous oxygen and ammonia on the ZnO surface, as per Equations (3) and (4), respectively. Due to its higher electron affinity, the molecular oxygen pinches the conduction band electrons and forms the adsorbed oxide ion. The two adjacent adsorbed species interact via hydrogen bonding (Equation (5)) to form the [NH_2_.H---O]^−^ complex, which further reacts with an additional adsorbed oxide ion as shown in Equation (6), releasing the captured electrons back into the conduction band, resulting in the observed decrease in the sensor film resistance.

**Figure 14.**
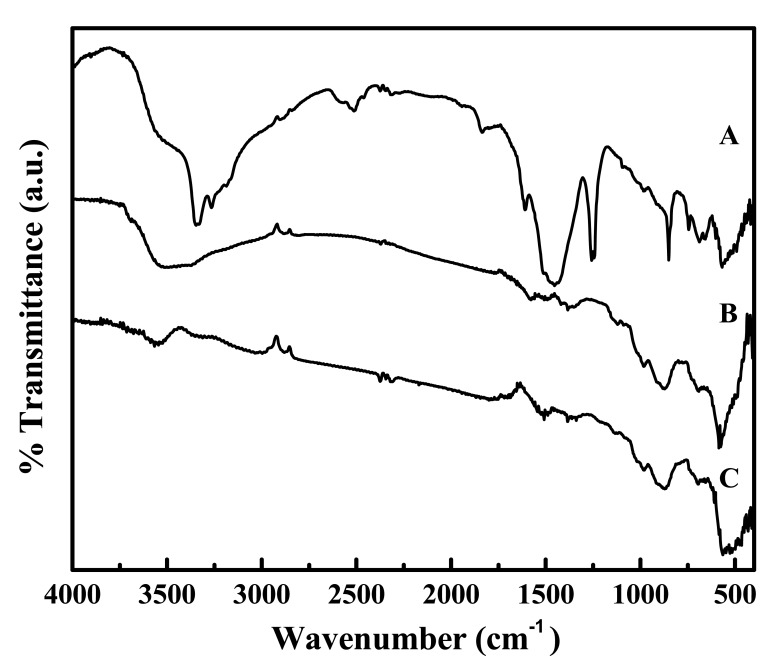
Comparative Fourier Transform Infrared (FTIR) signatures collected on the ZnO-based sensor film after: (**A**) exposing to 11,000 ppm ammonia for 40 min. at room temperature (23 °C), (**B**) recovery in dry air, and (**C**) heating 350 °C for 15 min.

The sensing pathway suggested above was supported by the comparative FTIR signatures, shown in Figure 14 after (A) its exposure to 11,000 ppm of ammonia at room temperature (23 °C), (B) recovery in dry air, and (C) heating for 15 min at 350 °C. The peaks appearing at 3,500–3,000, 1,605, 1,460 and 1,255 cm^−1^ in Figure 14(A) correspond to the vibration modes of ammonia adsorbed on the zinc oxide surface [39]. Upon recovery in dry air the peaks characteristic of N-H vibration disappeared whereas a wide band appeared in the range 3,600–3,000 cm^−1^. The latter peak corresponded to the adsorbed water molecules, which substantiated the formation of water in the reaction, described in Equation (6). Moreover, up on heating up to 350 °C, the sensor lost the adsorbed water, as indicated by the diminution of the water absorption band in its FTIR profile, displayed in C.

**Figure 15.**
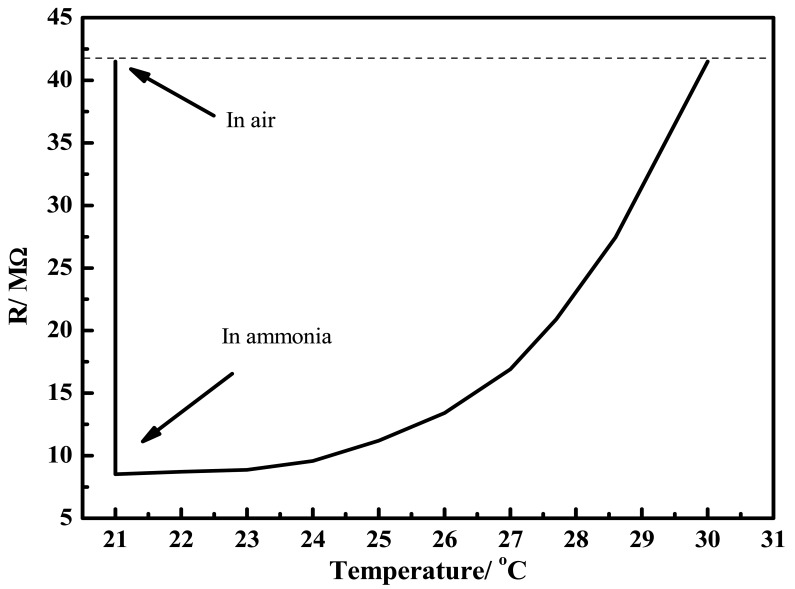
Variation in resistance of the ZnO sensor as a function of temperature in the presence of 590 ppm ammonia in the ambient.

The sensor response was also evaluated at different temperatures. In this case, the sensor was exposed to 590 ppm of ammonia in air, while the temperature of the chamber was increased slowly and the film resistance was monitored continuously. The temperature dependence of the sensor resistance in the presence of 590 ppm of ammonia, in the range of 21–30 °C is shown in Figure 15. Upon exposure to ammonia vapors at 21 °C, the resistance decreased immediately. Upon increasing the temperature at the same concentration of ammonia in air, the film resistance increased and reached an optimum value equal to that in ammonia-free air at 30 °C. Thus, the sensor performed well in the low temperature regime; at higher temperatures, desorption of ammonia and oxygen both from the ZnO surface predominates, thereby making the reactions shown in Equations (4) and (5) less probable and the film resistance gradually increases and eventually tallies that of the film in air.

## 4. Conclusions

Preparation of monoshaped and monosized particles of zinc basic carbonate and zinc oxide by the urea and hexamine-based homogeneous precipitation process, under boiling and sub-boiling conditions is described. Among various process variables, the temperature at which precipitation occurred proved to be the most important one. The particles formed under sub-boiling conditions were composed of zinc basic carbonate, while those precipitated under boiling conditions were composed of either a mixture of zinc basic carbonate and zinc oxide or pure zinc oxide. The as-prepared particles were thermally stable and retained their morphological artifacts on calcination at high temperature. These zinc oxide particles were used in the fabrication of sensors for the detection of ammonia at room temperature. These sensors showed long shelf life and reproducible performance, which we attribute to unique morphology of the zinc oxide particles produced in this work.

## Figures and Tables

**Table 1.** Preparation conditions for the particles displayed in various figures.

**Figure**	**Preparation Conditions**
Figure 1(A)	Aqueous solution, containing 0.4 mol/L urea and 0.035 mol/L zinc nitrate, and 10 vol.% ethylene glycol was heated for 45 min. at 80 °C.
Figure 1(B)	Aqueous solution, containing 0.4 mol/L urea and 0.035 mol/L zinc nitrate, and 10 vol.% ethylene glycol was heated for 45 min. at 95 °C.
Figure 2(A,B)	Aqueous solution, containing 0.4 mol/L urea, 0.035 mol/L zinc nitrate and 10 vol.% ethylene glycol was boiled under reflux for 50 min.
Figure 2(C)	Aqueous solution, containing 0.4 mol/L urea, 0.035 mol/L zinc nitrate and 10 vol. % ethylene glycol was boiled under reflux for 20 min.
Figure 2(D)	Boiling of solution obtained from 2C under reflux for 30 min.
Figure 3(A,B)	Aqueous solution, containing 0.4 mol/L urea, 0.035 mol/L zinc nitrate was boiled under reflux for 50 min.
Figure 3(C)	Aqueous solution, containing 0.4 mol/L urea, 0.035 mol/L zinc nitrate was boiled under reflux for 20 min.
Figure 3(D)	Boiling of solution obtained from 3C under reflux for 30 min.
Figure 4(A)	Reflux boiling of aqueous solution containing 0.035 mol/L zinc nitrate and 0.17 mol/L urea for 20 min.
Figure 4(B)	Reflux boiling of aqueous solution containing 0.035 mol/L zinc nitrate and 0.17 mol/L urea for 5 min.
Figure 5(A)	Reflux boiling of aqueous solution containing 0.034 mol/L zinc nitrate and 0.013 mol/L hexamine for 40 min. with 4.5 vol.% ethylene glycol.
Figure 5(B)	Reflux boiling of aqueous solution containing 0.034 mol/L zinc nitrate and 0.013 mol/L hexamine for 40 min. with 4.5 vol.% ethanol.
Figure 6(A)	Reflux boiling of aqueous solutions containing 0.2 mol/L urea, and 0.03 mol/L zinc nitrate for 30 min.
Figure 6(B)	Reflux boiling of aqueous solutions containing 0.4 mol/L urea, and 0.03 mol/L zinc nitrate for 30 min.
Figure 6(C)	Reflux boiling of aqueous solutions containing 0.4 mol/L urea and 0.03 mol/L zinc nitrate, with 15 vol.% ethylene glycol for 30 min.
Figure 6(D)	Reflux boiling of aqueous solutions containing 0.4 mol/L urea and 0.03 mol/L zinc nitrate, with 4 vol.% ethylene glycol for 30 min.
Figure 6(E)	Reflux boiling of aqueous solutions containing 0.4 mol/L urea and 0.015 mol/L zinc nitrate, with 4 vol.% ethylene glycol.for 30 min.
Figure 6(F)	Particles obtained by calcining the particles in 6D at 700 °C for 1 h (heating rate: 15°/min.)
Figure 9(A)	Zinc oxide particles obtained by calcining zinc basic carbonate 1B at 700 °C for 1 h
